# Supplementary figures and images for: CD300lf is the primary physiologic receptor of murine norovirus but not human norovirus
Source: PLoS Pathog. 2020 Apr 6;16(4):e1008242. doi: 10.1371/journal.ppat.1008242 (PMC7162533; doi:10.1371/journal.ppat.1008242)

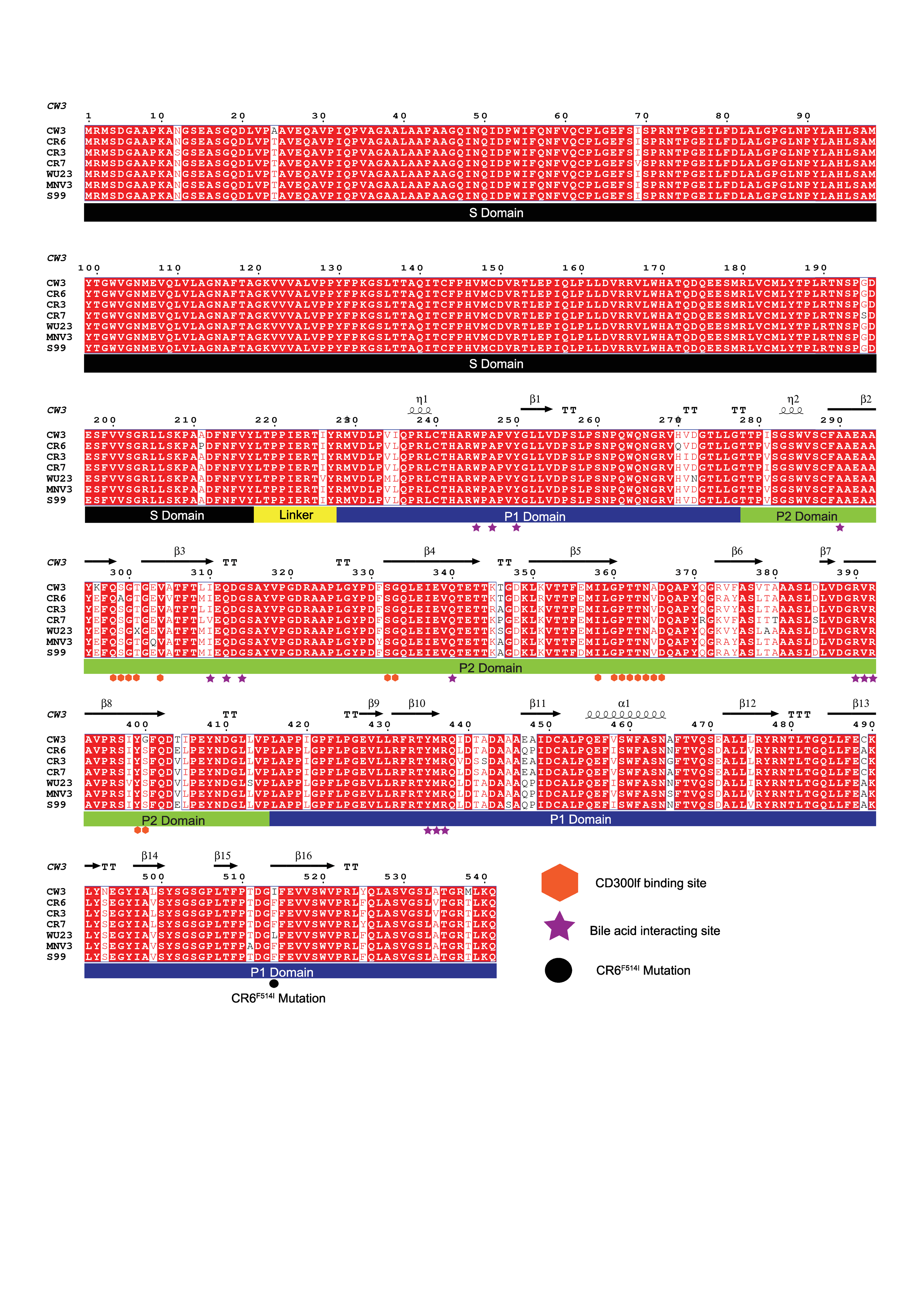

Supplement: S1 Fig — VP1 is the major structural protein of MNoV and is comprised of a shell and protruding domain. The complete VP1 sequence of MNoV strains CW3, CR6, CR3, CR7, WU23, MNV3, and S99 were aligned. The VP1 shell domain comprises the core of the virion and is sufficient for virion assembly. The protruding domain mediates binding to CD300lf and bile salts and is comprised of discontinuous P1 and P2 subdomains. The CD300lf and secondary bile acid (GCDCA) binding sites are highlighted as is the F514I mutation which emerged during infection of Cd300l+/-Stat1-/- and Cd300lf-/-Stat1-/- mice13. Secondary structures labeled as alpha-helices (α), 310-helices (η), and beta-strands (β). The number following the annotation is the numerical order of that secondary structure. Helices are displayed as squiggles and strands are represented by a forward moving arrow under the annotation. TT = strict β-turns and TTT = strict α turns. (TIF) [file ppat.1008242.s001.tif]
